# Relationships among Consumer Liking, Lipid and Volatile Compounds from New Zealand Commercial Lamb Loins

**DOI:** 10.3390/foods10051143

**Published:** 2021-05-20

**Authors:** Enrique Pavan, Yangfan Ye, Graham T. Eyres, Luis Guerrero, Mariza G. Reis, Patrick Silcock, Patricia L. Johnson, Carolina E. Realini

**Affiliations:** 1 AgResearch Limited, Te Ohu Rangahau Kai, Massey University Campus, Palmerston North 4442, New Zealand; Enrique.Pavan@agresearch.co.nz (E.P.); Yangfan.Ye@agresearch.co.nz (Y.Y.); Mariza.GomesReis@agresearch.co.nz (M.G.R.); 2 Departamento de Producción Animal, Estación Experimental Agropecuaria Balcarce, Instituto Nacional de Tecnología Agropecuaria, c.c. 276, Balcarce 7620, Argentina; 3 Animal Science, School of Agriculture and Environment, Massey University, Private Bag 11222, Palmerston North 4442, New Zealand; 4 Department of Food Science, University of Otago, P.O. Box 56, Dunedin 9054, New Zealand; graham.eyres@otago.ac.nz (G.T.E.); pat.silcock@otago.ac.nz (P.S.); 5 IRTA-Monells, Finca Camps i Armet, 17121 Monells, Spain; Lluis.Guerrero@irta.es; 6 AgResearch Invermay, Puddle Alley, Mosgiel 9092, New Zealand; Tricia.Johnson@agresearch.co.nz

**Keywords:** eating quality, fatty acids, flavor, meat, volatile compounds

## Abstract

Loin sections (m. *Longissimus lumborum*) were collected at slaughter from forty-eight lamb carcasses to evaluate consumer-liking scores of six types of typical New Zealand commercial lamb and to understand the possible underlying reasons for those ratings. A consumer panel (*n* = 160) evaluated tenderness, juiciness, flavor liking, and overall liking of the different types of lamb loins. Consumer scores differed among the types of lamb meat for all the evaluated attributes (*p* < 0.05). Further segmentation based on overall liking scores showed two consumer clusters with distinct ratings. Correlation and external preference map analyses indicated that one consumer cluster (*n* = 75) liked lamb types that had lower total lipid content, a lower proportion of branched-chain fatty acids, oleic and heptadecanoic acids; and a higher proportion of polyunsaturated fatty acids and volatile compounds (green and fruity descriptors). Consumer liking of the other segment (*n* = 85) was less influenced by fatty acids and volatiles, except hexanoic, heptanoic and octanoic acids (rancid, fatty, and sweaty descriptors). Thus, the fatty acid profile and the volatile compounds derived from their oxidation upon cooking seem to be a stronger driver of consumer liking of lamb for some consumers than others.

## 1. Introduction

Consistent and high-quality meat production that satisfies consumer expectations is of utmost importance to the meat industry to maintain and expand markets. A shift from traditional commodity to value-based marketing requires optimizing production and processing to guarantee quality and obtain a premium position in the market. To understand consumer perceptions of quality, it is necessary to characterize the eating quality of typical commercial lambs from different pasture-based production systems in New Zealand. The main sensory attributes of meat from a consumer perspective are tenderness, juiciness and flavor liking, which are influenced by several pre- and postharvest variables [1]. The preharvest variables that define raw material quality are animal genotype and gender, intramuscular fat (IMF) content, carcass muscling and fatness, and feeding systems [1]. All these factors influence the lipid composition of meat [2], which then influences the flavor through the production of various volatile compounds generated by oxidation upon cooking and their interaction with Maillard reaction products [3]. The extent of lipid oxidation is also affected by other factors, such as meat antioxidant and pro-oxidant status, which is also related to pre- and postharvest factors.

Meat flavor perception is also influenced by consumers’ previous experience and cultural background [4]. Thus, the country of origin of consumers has been reported to significantly affect their preferences for lamb meat, with consumers preferring forage or concentrate-fed lamb depending on their nationality [5]. Numerous studies have evaluated the effect of these two contrasting feeding systems on meat flavor and their relationship with levels of fatty acid and volatile compounds [6,7,8,9]. Although the impact that different pasture-based production systems have on lamb meat fatty acid profiles has been characterized [10,11,12,13], less is known about its impact on eating quality. Lamb production in New Zealand is mainly based on grazing systems where various pasture combinations suit different environments across the country. Different pasture systems may also result in lambs with different carcass weight and in meat IMF content, color and shear force [14]. Phelps et al. [15] recently reported that consumer overall liking and flavor-liking scores differed between lamb meat from United States, Australia and New Zealand. Moreover, consumer scores also differed when comparing New Zealand lamb meat obtained from different seasons and regions [16].

The main objectives of the present study were to evaluate consumer-liking scores for different types of typical New Zealand commercial lamb and to understand the underlying reasons for those ratings by looking at the association between consumer-liking scores with lipid content and fatty acid profile from raw meat and with volatile compounds from cooked meat.

## 2. Materials and Methods

### 2.1. Lamb Samples

Loin sections (m. *Longissimus lumborum*) were collected at slaughter from forty-eight lamb carcasses from six typical New Zealand commercial animal groups that differed in age at harvest, genetics, sex, and the type of pasture they grazed at finishing. Animals represented forage-fed early, mid and late-season lambs typically processed in New Zealand. These included WEAN-W: 4 month-old wether lambs of composite genetics (Perendale, Texel, Finnish Landrace and Romney) slaughtered at weaning (suckled and grazing mothers’ diet of a chicory and red clover mix), REDC-W: 6–8 month-old wether lambs of composite genetics (Perendale x LambSupreme) that had been grazing red clover, GRASS-W: 6–8 month-old wether lambs of composite genetics (Perendale, Texel, Finnish Landrace and Romney) that had been grazing a predominantly Italian and perennial ryegrass pasture, CHIC-E and CHIC-W: 6–8 month-old ewe and wether lambs of composite genetics (Perendale, Texel, Finnish Landrace and Romney) that had been grazing chicory, and PMER-W: 12 month-old Merino wether lambs that had been grazing a mixed pasture (mainly perennial ryegrass and white clover mix). A detailed description of animal groups, sample collection and carcass and meat quality characteristics corresponding to this study were previously reported by Ye, Schreurs, Johnson, Corner-Thomas, Agnew, Silcock, Eyres, Maclennan and Realini [14], as well as the fatty acid composition and volatiles from raw meat [13]. In the present study, volatile compounds from cooked meat are presented and discussed. Collected lamb samples were kept vacuum-packed at −1.5 °C for 21 days, followed by storage at −20 °C until further analysis.

### 2.2. Consumer Sensory Evaluation

One hundred and sixty consumers were recruited in Dunedin (New Zealand) in June 2018 to quantify the overall liking, flavor liking, tenderness and juiciness of the six lamb types and to determine the willingness to pay across four conceptual levels of lamb quality [17,18]. Vacuum-packed loin sections (approximately 15 cm) were thawed at 4 °C for 24 h and then cooked using sous vide at 57 °C for 1 h. After cooking, the loins were removed from the sous vide bag, dried using paper towels and rested at room temperature for 3 min. After resting, loins were grilled on a 170 °C hot plate grill (Blue Seal Evolution Series EP516 electric griddle; Moffat Limited Christchurch, New Zealand) for 5 min with the fat side down, and then 3 min on the other side to obtain a core temperature of 60 °C (medium degree of doneness). After cooking, loins were rested for 3 min and then trimmed of fat and placed at 40 °C in a Bain Marie to keep warm before serving. The maximum holding time was 50 min. Lamb loin samples were sliced to 0.8 cm thickness (to obtain approximately 20 portions per loin) using a cutting guide and served on demand to consumers with a random 4-digit code. Seven loin samples (one warmup sample and six evaluation samples) were presented monadically to consumers. The warmup sample was presented first and was from the same loin for all 20 consumers within the tasting session. To minimize first-order and carryover effects, the remaining six samples were presented to consumers in order following a Williams Latin square design [19]. The evaluation of the samples was performed using portable divisions for consumer testing in a room with controlled environmental conditions under white light. A total of 8 tasting sessions were carried out with 20 consumers per session. Consumers were given plain water, diluted apple juice and crackers to cleanse their palate between samples. Each sample was rated on four 100 mm nonstructured line scales for overall liking, flavor liking, juiciness and tenderness, anchored at 5 mm with either (0: dislike extremely, not juicy, not tender to 100: like extremely, very juicy, very tender). Consumers also indicated their willingness to pay (NZD per kg) for the following categories of lamb quality: unsatisfactory, good everyday, better than everyday and premium [17].

### 2.3. Fatty Acid Analysis

The total and individual fatty acid content of raw meat were determined using a gas chromatography (GC) method according to Agnew et al. [20]. Detailed sample preparation and analytical conditions used were previously reported by [13]. Total fatty acid content is express in mg/100 g of raw meat and individual fatty acids as the proportion of total fatty acid content.

### 2.4. Volatile Compound Analysis

Vacuum packed sections (~35 mm) of frozen lamb loins were defrosted at 4 °C for 24 h before testing. One loin from each of the 6 animal groups was randomly chosen on each analysis day (8 days total). Loin sections were cooked following the same protocol as for the consumer panel. After cooking, samples were cooled at room temperature, vacuum-packed in foil laminate bags, then snap-frozen with liquid N2 for 5 min (semi-frozen) and transferred to a freezer −20 °C for ~1 h before sample preparation. Samples were frozen to facilitate the core sample collection, which enabled a controlled mass and sample composition for volatile analysis. It also minimized any potential loss or change of volatile compounds during preparation.

For each cooked loin section, two analytical replicates were prepared for analysis. For each sample replicate, three-cylinder pieces (5 mm diameter × 15 mm) were cut using a stainless-steel corer (parallel punch to fat cap). The cooking surfaces of the cylinder pieces were removed from the ends to achieve ~15 mm sections and adjusted as necessary to achieve the target weight (4.0 ± 0.1 g). The three-cylinder pieces were then added to a 20 mL glass headspace vial. A 250 μL insert tube was positioned vertically inside the vial, and 50 μL of an internal standard (1.25 mg/L fenchol, 99% purity, Sigma-Aldrich, Madrid, Spain) was added using an HPLC syringe. Samples were kept at 4 °C until analysis (maximum 12 h). Vials were transferred to an autosampler tray (PAL3 RSI 85, CTC Analytics, Zwingen, Switzerland) for analysis. To minimize the chance of volatile compound changes, only 4 vials were transferred at a time so that samples were held for no longer than 2.5 h in the autosampler tray. The room temperature was kept at 18 °C.

Volatile compounds were extracted using solid-phase microextraction (SPME) using 50/30 μm divinylbenzene/carboxen/polydimethylsiloxane (DVB/CAR/PDMS) fiber (Supelco, Bellefonte, USA). Vials were equilibrated at 37 °C for 5 min before extracting the headspace of the unstirred sample for 30 min. The SPME fiber was then desorbed directly in the injection port of the chromatographic system under the conditions detailed below. After desorption, the fiber was cleaned for 2 min at 270 °C before the subsequent extraction.

Samples were prepared for analysis according to a predetermined order using a randomized complete block design to limit first-order and carryover effects, also blocked by sample replicate (2 replicates per loin section). A randomly selected animal from each of the six treatments was prepared each day, such that the analyses were conducted over an 8 d period.

Gas chromatography-mass spectrometry (GC–MS) was conducted using an Agilent Technologies 6890 N GC (Beijing, China) equipped with an Agilent 5975 B, VL MS triple axis detector (Wilmington, DE, USA). The fiber was desorbed directly in the injection port of the GC–MS at 240 °C for 5 min; 2 min in splitless mode followed by 3 min with a purge flow of 60 mL/min. Helium was the carrier gas at a flow rate of 1.2 mL/min. A ZB-WAX capillary column (Phenomenex, Torrance, CA, USA) of 60 m × 0.32 mm I.D. × 0.5 µm film thickness was used for separations. The oven temperature was initially 50 °C for 2 min, then increased at 10 °C/min to 240 °C and held at this temperature for 10 min. The mass spectrometry parameters were a transfer line temperature of 200 °C; quadrupole temperature at 150 °C with an emission current of 35 μA; and an ion source at 230 °C. The runtime was recorded in full scan mode (*m*/*z* 29–300 mass range). The chromatographic data were analyzed by Masshunter^®^ (version B.07.02, Agilent Technologies, Santa Clara, CA, USA). The raw GC–MS data were exported in CDF format. The deconvolution of peaks was performed using the PARAFAC2-based deconvolution and identification system (PARADISe), version 3.2 (University of Copenhagen, Denmark) [21]. The relative concentrations of the deconvoluted compounds were extracted for further analysis. Tentative identification of compounds was obtained by comparing the deconvoluted mass spectra and calculated retention indices against the National Institute of Standards and Technology NIST14 GC–MS database. The linear retention index (RI) of each peak was calculated using a C7–C30 saturated alkane standard (Supelco, Bellefonte, PA, USA) using the same GC temperature program.

### 2.5. Statistical Analysis

Differences between commercial lamb-meat products (product) for tenderness, juiciness, flavor liking, and overall liking scores were evaluated as a completely randomized design by ANOVA using XLSTAT 2017 (Addinsoft 2012) software (Addinsoft, Paris, France). The model included product as a fixed effect and consumer (All consumers) as a random effect. An agglomerative hierarchical cluster analysis was performed on the square Euclidean distance matrix, with the Ward method, to identify two clusters of consumers based on their normalized overall liking scores (cluster 1 and cluster 2) using XLSTAT. Consumer panel data were then analyzed as above, but considering consumers by cluster as a random effect. Significance was declared at *p* < 0.05 unless otherwise noted.

Product effects on the relative abundance of volatile compounds were evaluated as a completely randomized design by ANOVA using the mixed procedure in SAS (SAS University Edition, SAS Institute Inc., Cary, NC, USA). The model included product and block (analysis day) as fixed effects. Some variables were transformed (Log10 or 1/x) to reach a normal distribution, and results were back-transformed for presentation.

Relationships between consumer scores from all consumers, consumers in cluster 1 and cluster 2, fatty acid profile, and volatile compounds were assessed using the correlation (CORR) procedure in SAS to generate Pearson’s correlation coefficients. The regression (REG) procedure in SAS was used for multivariate regression analysis, using the stepwise selection option, with fitted variables required to be significant (*p* < 0.05) to remain in the final model. The associations between consumer liking of commercial lamb-meat products and fatty acid profile and volatile compounds were further evaluated through their representation in an external preference map. Therefore, commercial lamb-meat products, fatty acid profile, and volatile compounds correlated with flavor liking or overall liking scores from consumers in cluster 1 and cluster 2 were subjected to principal component analysis (PCA) using the XLSTAT 2017. The PREFMAP procedure from XLSTAT 2017 was applied. Product evaluations for each cluster were modeled using product characteristics as explanatory variables. To remove the effect of scale between variables in the PCA, Pearson’s correlation coefficients were used as an index of similarity.

Willingness to pay for each of the four lamb quality categories was analyzed using the GLIMMIX procedure from SAS. Meat quality grade (MQ) was included as a fixed effect in the model. The homogeneity of variance between MQ was evaluated using the COVEST HOMOGENEITY statement. To account for the lack of homogeneity in the model, the statement “RANDOM_residual_/GROUP = MQ” was used.

## 3. Results

When considering the responses from all consumers, scores significantly differed between the six types of commercial lamb for tenderness, flavor liking and overall liking (*p* ≤ 0.001; Table 1). Tenderness scores were higher (*p* < 0.05) for REDC-W, CHIC-E and CHIC-W compared with PMER-W, while GRASS-W and WEAN-W were similar to the other groups. Flavor-liking scores were higher (*p* < 0.05) for REDC-W and CHIC-W compared with WEAN-W and PMER-W, while CHIC-E and GRAS-W were similar to the other groups. The overall liking scores for CHIC-W were higher (*p* < 0.05) than PMER-W and WEAN-W. GRASS-W and CHIC-E did not differ (*p* > 0.05) in overall liking scores from the other groups, whereas scores from REDC-W were higher (*p* < 0.05) than those from PMER-W.

Two clusters of consumers were identified based on how they scored the overall liking of meat from the six different commercial animal groups. About half of the consumers (cluster 1, *n* = 85) overall preferred (*p* < 0.05) meat from GRASS-W than from WEAN-W, REDC-W and CHIC-E, while meat from CHIC-W and PMER-W were similar (*p* > 0.05) to that from GRASS-W, WEAN-W and REDC-W. In contrast, overall liking scores assigned by consumers in cluster 2 (*n* = 75) were higher (*p* < 0.05) for meat from CHIC-E, REDC-W and CHIC-W than for GRASS-W and PMER-W, while meat from WEAN-W was similar (*p* > 0.05) to that from CHIC-W, GRASS-W and PMER-W. It should be noted that meat from CHIC-W was highly rated by both consumer segments, resulting in the highest overall consumer score (*n* = 160). In cluster 1, tenderness scores did not differ (*p* > 0.05) between the different types of lamb. In contrast, scores for juiciness were higher (*p* < 0.05) for GRASS-W than CHIC-E and REDC-W. Scores for flavor liking were higher (*p* < 0.05) for GRASS-W than WEAN-W, REDC-W and CHIC-E, which did not differ (*p* > 0.05), while CHIC-W liking scores were similar (*p* > 0.05) to GRASS-W and to REDC-W scores, and PMER-W was similar (*p* > 0.05) to all other treatments. In cluster 2, the tenderness scores for CHIC-E, REDC-W and CHIC-W were higher (*p* < 0.05) than GRASS-W and PMER-W, while WEAN-W tenderness scores were similar (*p* > 0.05) to CHIC-W, GRASS-W and PMER-W. Juiciness scores for CHIC-E and REDC-W were higher (*p* < 0.05) than PMER-W, while CHIC-W, WEAN-W and GRASS-W did not differ (*p* > 0.05) from the other groups. Flavor-liking scores were higher (*p* < 0.05) for REDC-W, CHIC-E and CHIC-W than GRASS-W and PMER-W. In contrast, WEAN-W scores were similar (*p* > 0.05) to CHIC-W and to GRASS-W. As the sociodemographic and behavioral variables of the consumers did not differ (*p* > 0.05) between the two clusters, no further analysis was carried out.

All consumer scores for tenderness, juiciness and flavor liking were positively correlated with overall liking scores (*p* < 0.001). The highest correlation was with flavor liking (*r* = 0.91), followed by tenderness (*r* = 0.71) and juiciness (*r* = 0.66). When a stepwise regression approach was used to determine the most important contributing variables, 87.2% of the variation in overall liking was explained by tenderness, juiciness, and flavor-liking scores (*p* < 0.001; MSE 49.7), with 82.3% explained by flavor liking. Variation in the tenderness and juiciness scores contributed to overall liking by 4.0% and 0.9%, respectively. The final overall liking model showed that consumer overall liking rating = 1.77 + (0.18 × Tenderness) + (0.11 × juiciness) + (0.69 × flavor liking). Significant correlations were found between the consumer scores from each cluster for tenderness (*r* = 0.54, *p* < 0.001) and juiciness (*r* = 0.34, *p* < 0.01), but no correlations were found for flavor and overall liking (*p* > 0.10), indicating that the two clusters rated liking of the lamb samples differently.

Correlations of overall liking scores with total fatty acid content and proportions of selected fatty acids in fresh meat for all consumers and consumers in cluster 1 and cluster 2 individually are shown in Table 2. Total *Longissimus* muscle fatty acid content (mg/100 g fresh tissue) was negatively correlated (*r* = −0.34, *p* < 0.05) with overall liking scores from cluster 2, but no correlation was observed with scores from all consumers or scores from cluster 1 consumers (*p* > 0.05). Overall liking scores from cluster 2 consumers were also correlated (*p* < 0.05) with the proportion (% of total fatty acids) of specific fatty acids or classes of fatty acids in meat, whereas only a few trends (*p* < 0.10) were observed when considering all consumers or cluster 1 consumers scores. The proportions of individual or classes of fatty acids that were correlated with overall liking scores from cluster 2 consumers were also correlated with a total fatty acid content, but in the opposite direction (positive instead of a negative correlation and vice versa) except for iso-C17:0 and C17:1 that were not correlated. In general, the absolute value of cluster 2 overall liking score correlation coefficients with the proportions of individual or group of fatty acids were higher than with total fatty acid content. Correlations were negative between consumer overall liking scores and individual (iso-C15:0, iso- and anteiso-C17:0) and total branched-chain fatty acids (BCFA), total saturated fatty acids (SFA) and total mono-unsaturated fatty acids (MUFA) as well as with C17:0 and C18:1 cis-9. They were positive with total polyunsaturated fatty acids (PUFA), total *n-6*, total *n-3*, individual PUFA (C18:2 *n-6*, C18:3 *n-3*, C20:5 *n-3*, C22:5 *n-3* and C22:6 *n-3*), C17:1 cis-10, C18:1 cis-11 and trans-11 from cluster 2. Similar correlations were observed between consumer flavor-liking scores and fatty acid profile, with only slightly higher absolute values for the correlation coefficients than overall liking scores.

Fifty-five volatile compounds were identified in cooked lamb meat and classified according to their chemical structure (Table 3). The relative abundances of these compounds in the six types of cooked lamb are presented in Table 4. Twenty-five of the identified volatile compounds differed among the lamb meat types (*p* < 0.05), with thirteen of those compounds having literature odor descriptors, including green or fruity characters (5 alcohols: 1-pente*n-3*-ol, Z-2-penten-1-ol, 1-octe*n-3*-ol, 1-heptanol and E-2-octen-1-ol; 4 aldehydes: pentanal, hexanal, E,E-2,4-heptadienal and E-2-nonenal; 1 benzenoid compound: benzaldehyde; 1 furan: 2-pentyl furan; 1 hydrocarbon: E-2-octene; and 1 ketone: 6-methyl-5-hepten-2-one) [22,23,24,25,26]. In general, meat from WEAN-W showed a higher (*p* < 0.05) abundance of all these compounds than PMER-W, except for benzaldehyde and pentanal, which did not differ (*p* > 0.05). More specifically, the abundance of 1-pente*n-3*-ol was highest in WEAN-W and higher in CHIC-E than GRASS-W and PMER-W. At the same time, REDC-W and CHIC-W were similar (*p* > 0.05) to CHIC-E, GRASS-W, and PMER-W. Similarly, the abundance of Z-2-penten-1-ol was highest in WEAN-W. CHIC-E showed a higher abundance of Z-2-penten-1-ol than GRASS-W and PMER-W and CHIC-W higher than PMER-W, while REDC-W did not differ from the other groups except WEAN-W. The abundances of 1-octe*n-3*-ol and E-2-octen-1-ol were higher (*p* < 0.05) in WEAN-W than in REDC-W, GRASS-W and PMER-W. In contrast, the abundance of 1-Heptanol was higher (*p* < 0.05) in WEAN-W, REDC-W, GRASS-W, CHIC-E and CHIC-W than in PMER-W. The abundance of pentanal was higher in CHIC-W than RED-W, GRASS-W and PMER-W, and also higher in CHIC-E, WEAN-W and RED-W compared with PMER-W (*p* < 0.05). The abundance of pentanal in GRASS-W did not differ (*p* > 0.05) from PMER-W. Hexanal abundance was also higher (*p* < 0.05) in WEAN-W, CHIC-E and CHIC-W than in PMER-W, while PMER-W did not differ (*p* > 0.05) from RED-W and GRASS-W. E,E-2,4-Heptadienal abundance was higher (*p* < 0.05) in WEAN-W and CHIC-W than in REDC-W, GRASS-W and PMER-W, while CHIC-E did not differ (*p* > 0.05) from REDC-W, GRASS-W, CHIC-W or PMER-W. The abundance of E-2-nonenal was similar (*p* > 0.05) in WEAN-W, REDC-W, CHIC-E and CHIC-W, while the abundance was higher (*p* < 0.05) in WEAN-W and CHIC-W than PMER-W. Benzaldehyde was higher (*p* < 0.05) in CHIC-E and CHIC-W than GRASS-W and PMER-W. 2-Pentyl-furan was higher in WEAN-W, CHIC-E and CHIC-W than in PMER-W. The abundance of E-2-Octene was highest in WEAN-W. The abundance of this volatile was also higher (*p* < 0.05) in PMER-W than CHIC-E and CHIC-W, while REDC-W and GRASS-W did not differ (*p* > 0.05) from the other groups except WEAN-W. The abundance of 6-methyl-5-hepten-2-one was higher (*p* < 0.05) in WEAN-W, CHIC-E and CHIC-W than PMER-W and GRASS-W. The abundance of butanoic acid was higher (*p* < 0.05) in WEAN-W, REDC-W, CHIC-E and CHIC-W than in PMER-W.

The abundance of three identified sulfur volatile compounds that share sulfurous literature odor descriptors [23,24,25] differed among the types of lamb meat (*p* < 0.05). The abundance of dimethyl sulfide was higher in WEAN-W than REDC-W, GRASS-W and PMER-W and was also higher in CHIC-E and CHIC-W than GRASS-W. The abundance of 2-ethyl-1-hexanethiol was highest in WEAN-W. The abundance of this volatile was also higher in CHIC-W than PMER-W, while REDC-W, GRASS-W and CHIC-W only differed from WEAN-W. The abundance of dimethyl sulfone was higher in WEAN-W, REDC-W and PMER-W than in CHIC-E and CHIC-W, and it was also higher in REDC-W than GRASS-W. The abundance of 1-butanol, which reportedly has a fusel odor character, was higher in PMER-W and WEAN-W than in the other lamb types (*p* < 0.05). The abundance of pentadecanal (fresh and waxy) was higher in WEAN-W, REDC-W, CHIC-E and CHIC-W than in GRASS-W and PMER-W. The abundance in GRASS-W was also higher than in PMER-W.

Overall liking scores from all consumers were correlated (*p* ≤ 0.05) with four volatile compounds (3 positively and 1 negatively), three were negatively correlated (*p* ≤ 0.05) with scores from cluster 1 consumers, and 12 compounds were correlated (11 positively and 1 negatively, *p* ≤ 0.05) with overall liking scores from cluster 2 consumers (Table 4). Six of the volatile compounds that were positively correlated with the overall liking scores from cluster 2 consumers had green, fruity, or fresh as literature odor descriptors (pentanal, heptanal, pentadecanal, benzaldehyde, 6-methyl-5-hepten-2-one, butanoic acid), while three compounds had sweet as a literature odor descriptor (2-ethyl-furan, acetoin, dimethyl sulfide). Overall, scores from cluster 1 consumers were negatively correlated with the abundance of 1-hexanol and E-2-nonenal, both with a green odor descriptor, and also negatively correlated with the abundance of octanoic acid, described as having a fatty, rancid odor. Of these compounds that were significantly correlated with consumer overall liking scores, not all were significantly different (*p* > 0.05) between meat types. In addition, not all volatile compounds that differed significantly (*p* < 0.05) between types of lamb meat were correlated with consumer overall liking scores.

The correlations between flavor liking consumer scores and the abundance of volatile compounds were very similar to those found between overall liking scores and volatiles, with a few more compounds showing correlations (*p* ≤ 0.05). Flavor-liking scores from cluster 2 consumers were positively correlated (*p* ≤ 0.05) with two other compounds with green odor descriptors (1-hexanol, *r* = 0.30, and E-2-nonenal, *r* = 0.36). In contrast, scores from cluster 1 consumers were negatively correlated to two other compounds with sour, fatty, sweaty, rancid and cheesy odor descriptors (hexanoic acids, *r* = −0.31; and heptanoic acid, *r* = −0.30).

**Figure 1:**
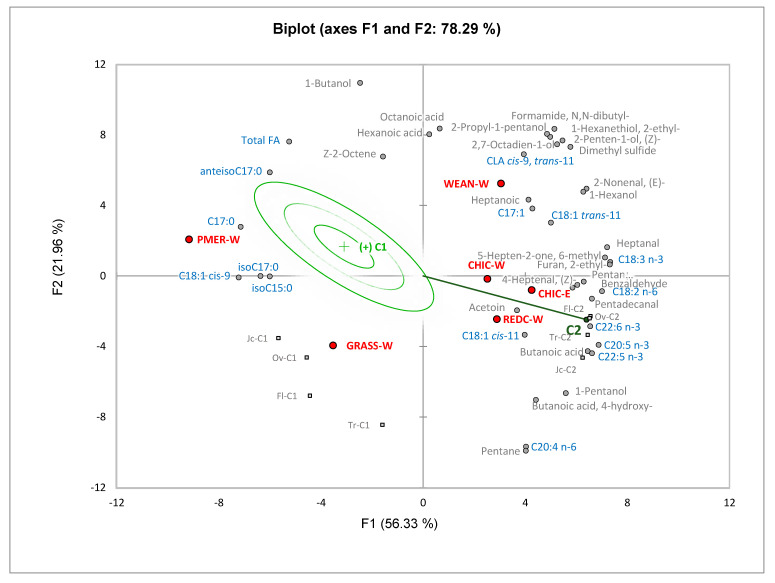
Cluster 1 and cluster 2 consumer preference map for fatty acids and volatile compounds. The ideal elliptical and vector models were significant (*p* = 0.005 and *p* =0.091 for cluster 1 and cluster 2, respectively). Ranks of preference (from most to least preferred) cluster 1: GRASS-W, CHIC-W, PMER-W, WEAN-W, REDC-W, CHIC-E; cluster 2: CHIC-E, REDC-W, CHIC-W, WEAN-W, GRASS-W, PMER-W. Supplementary variables (square symbols): tenderness (Tr-), juiciness (Jc-), flavor liking (Fl-) and overall liking (Ov-) for cluster 1 (C1) and cluster 2 (C2).

An external preference map (Figure 1) was obtained by joint analysis of meat composition data with consumer liking data. Chemical variables (total fatty acid content, proportions of 15 fatty acids and abundances of 24 volatile compounds) that were correlated (*p* ≤ 0.10) with overall or flavor-liking scores were included in the analysis. A two-dimension solution (78.29% of variance) was selected to display the result using vectorial and elliptical models. Separation on Factor 1 (56.33% of the variance) was due to differences in the fatty acid profile and volatile compounds. More specifically, PUFA were positively correlated with the volatile compounds derived from their oxidation and negatively correlated with mono-unsaturated fatty acids (MUFA), branched-chain fatty acids (BCFA) and total fatty acid content. Factor 2 (21.96% of variance) suggests a positive association between total fatty acids and volatile compounds with oily/fatty odor descriptors (1-butanol) or fatty and urine- and mutton-like odor descriptors (hexanoic and octanoic acids). Factors 3 and 4 combined explained a further 18.50% of the total variance but did not contribute any further insights to explain consumer liking of the different lamb products (*p* > 0.70, data not shown). Overall liking from cluster 1 consumers is described by an ideal point elliptical model (*p* = 0.005), whereas overall liking from cluster 2 consumers is represented by a vector model (*p* = 0.091). Cluster 1 consumers (*n* = 85) preferred lamb meat with higher proportions of BCFA, oleic acid and total fatty acids. In contrast, cluster 2 consumers preferred products with higher proportions of PUFAs that, upon cooking, generate a greater abundance of volatile compounds with green or fruity odor descriptors. According to consumer scores, the ranking of preferred types of lamb from highest to lowest was GRASS-W, CHIC-W, PMER-W, WEAN-W, REDC-W, and CHIC-E for cluster 1 consumers, and CHIC-E, REDC-W, CHIC-W, WEAN-W, GRASS-W, and PMER-W for cluster 2 consumers. The types of lamb meat preferred by cluster 2 consumers were positioned on the positive F1 axis, opposite from GRASS-W and PMER-W. Both clusters are associated with lamb types that are distanced from volatile compounds with oily, fatty, urine-like or mutton-like odor descriptors, indicating a lower liking of meat with high proportions of these compounds.

**Figure 2:**
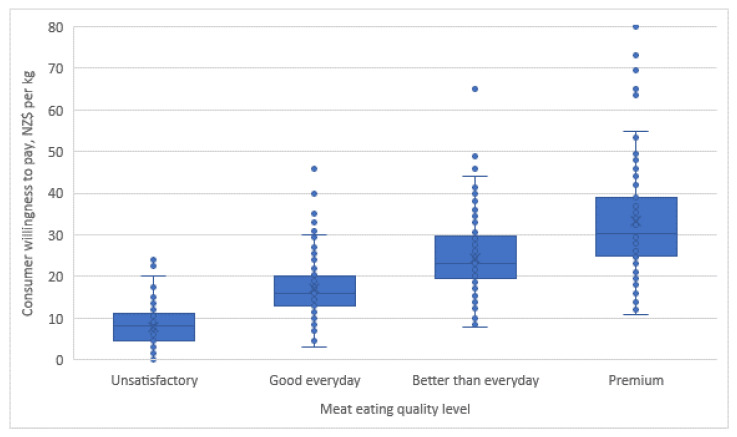
Box-plot of consumer willingness to pay (0−80 NZD per kg) of New Zealand consumers for each quality level of lamb loins. Least-square means from all meat-eating quality levels were different from each other (*p*-value < 0.001; unsatisfactory, NZD 8.0 ± 0.4; good everyday, NZD 17.1 ± 0.5; better than everyday, NZD 24.4 ± 0.7; premium, NZD 33.3 ± 1.0. Likelihood ratio test of the homogeneity of variances over the fixed effect of meat-eating quality, *p* < 0.001).

Figure 2 shows how much consumers were willing to pay for different levels of lamb eating quality. Consumer response was similar for both clusters, and therefore, data are presented for all consumers only. Consumer willingness to pay for lamb increased linearly with meat quality level (*p* < 0.001) with larger price variation for “premium” than “unsatisfactory” quality.

## 4. Discussion

The main objectives of the present study were to evaluate consumer-liking scores of different types of typical New Zealand commercial lamb and to understand the underlying reasons for those ratings by looking at the association between consumer-liking scores and lipid content, fatty acid profile and the level of volatile compounds in cooked lamb. Our results indicate that the overall liking for different types of commercial lamb differs among New Zealand consumers. They are willing to pay significantly higher prices for higher meat quality, showing a clear differential price opportunity for premium lamb. Consumer segmentation based on overall liking scores showed two clusters with distinct liking ratings of the different types of lamb, mainly driven by meat flavor. However, consumer clusters were not differentiated by their sociodemographic characteristics.

Similarly, when analyzing a larger data set (24,840 consumers), Thompson et al. [27] observed that flavor liking and overall liking were influenced by consumer appreciation of meat and preferred level of doneness, but not by any other demographic factor. They concluded that demographic factors had only a minor impact on sensory scores, making it difficult to identify a niche market for a given lamb product. As such, it is important to have a quality label as a reference for consumers to infer the expected meat quality based on their previous experience.

In agreement with results from other authors [15,27,28], flavor liking was the major driver of lamb meat overall liking, followed by tenderness and juiciness. In general, the level of tenderness across the loin samples in this study was rated as high, supporting the lower impact of tenderness relative to flavor on overall liking scores. A trend across different studies, including this one, are the high correlations observed among tenderness, juiciness and flavor liking. When tenderness is the main driver of overall palatability, it is relatively easy to identify the top or bottom quality products. Even in this study in which tenderness was not the major contributor to explaining overall liking, there was a general agreement among consumers from cluster 1 and cluster 2 for tenderness. In contrast, there was no agreement among consumers for flavor liking of the lamb meat types. This illustrates how cluster analysis can be a valuable tool in understanding drivers of overall liking of meat by allocating consumers with similar liking patterns into segments [29,30,31,32].

CHIC-W was assigned the highest overall liking scores by all consumers and was scored above-average in both clusters. In contrast, meat from GRASS-W and CHIC-E rated average by all consumers. Still, it had among the highest scores for consumers in cluster 1 and cluster 2, respectively. That neither flavor nor overall liking scores from consumers in cluster 1 were correlated with scores in cluster 2 suggests that differences in meat flavor between consumers are quite complex and difficult to predict.

The main contributors to meat flavor are the volatile compounds generated by lipid degradation and Maillard reactions of soluble components during cooking or through reactions between them [33]. Different studies have reported a positive relationship between overall liking and intramuscular fat or total muscle fat content in lambs [30,34,35,36]. These reports contrast with the lack of correlation between intramuscular fat and overall liking observed in the present study for all consumers and for consumers grouped in cluster 1 and the negative correlation observed between overall liking and intramuscular fat in cluster 2 (Table 2). The relatively small intramuscular fat content variation between samples could in part explain the lack of correlation observed with all consumers and consumers from cluster 1. The negative correlation between total fatty acids and overall liking scores for consumers in cluster 2 may be due to specific individual or classes of fatty acids than total fatty acids on consumer liking, especially at the low levels of intramuscular fat in the meat from this study. According to Savell and Cross [37], meat tenderness and juiciness are influenced by intramuscular fat. Still, the flavor is mainly influenced by the fatty acid profile. Pannier, Gardner, O’Reilly, and Pethick [1] suggested that a positive impact of intramuscular fat on meat palatability is due to its effect on tenderness when intramuscular fat content is high (>8%). Still, it is mainly due to flavor and juiciness when intramuscular fat levels are low. As observed in the present study and in others [38,39], intramuscular fat content is highly correlated with the fatty acid profile in meat. Thus, intramuscular fat and fatty acid composition effects on flavor are mostly confounded. In our study, the higher liking scores for leaner lamb meat in cluster 2 could be associated with its greater proportion of poly-unsaturated fatty acids and the volatiles they produce (1-Pentanol, 1-Hexanol, Pentanal, Heptanal, Z-4-Heptenal, E-2-Nonenal), and its lower proportion of oleic acid in the raw meat. In fact, overall liking and flavor-liking scores had higher correlations with total and individual fatty acid proportions than with total fatty acid content. The preference map shows that the proportions of PUFA and oleic acid are more important than total fatty acid content in the definition of cluster 2 overall liking. Karamichou et al. [40] and Realini, Pavan, Johnson, Font, Jacob, Agnew, Craigie and Moon [30] also observed strong and modest correlations between flavor-liking scores and fatty acid profile. However, they observed negative correlations with PUFA and positive correlations with MUFA concentrations. According to Font-i-Furnols and Guerrero [4], flavor preferences and acceptability of lamb meat are highly influenced by consumers’ consumption habits. For instance, Sañudo, Alfonso, San Julián, Thorkelsson, Valdimarsdottir, Zygoyiannis, Stamataris, Piasentier, Mills, Berge, Dransfield, Nute, Enser and Fisher [5] distinguished two groups of consumers based on their lamb flavor preferences that were highly associated with their origin. Consumers with a Mediterranean origin preferred milk- or concentrate-fed lamb. In contrast, consumers with a Northern origin preferred grass-fed lambs. New Zealand consumers are accustomed to consuming meat from pasture-fed animals. As a result, they may prefer the flavor of this type of product over that of concentrate-fed animals [41]. Feeding grass or concentrate to the animals changes the proportions of *n-3* and *n-6* PUFA in their muscles and, hence, the volatile compounds resulting from their oxidation, which have been associated with consumer preferences for lamb [42]. Meat flavor from concentrate-fed animals was also associated with greater proportions of oleic acid [3]. As lambs were always grazing pastures in the current study, the relationship between *n-3* and *n-6* fatty acids did not change, but cluster 2 consumers preferred meat with lower percentage of oleic acid, greater proportions of both *n-3* and *n-6* PUFA, and the volatile compounds that provided a green and/or fruity odor descriptors. It must be noted that the proportions of *n-3* and *n-6* PUFA were highly correlated, therefore, their relative proportions did not change significantly in the present study, as commonly happens when comparing meat from grass- and concentrate-fed animals. In agreement with our observations, Prescott et al. [43] observed that flavor descriptors, such as green, sweet and sheep-meat had a positive impact on meat liking assessed by New Zealand and Japanese consumers.

It has also been suggested that heptadecanoic acid (C17:0) could be used as a marker for volatile branched-chain fatty acids (vBCFAs: 4-methyloctanoic, 4-ethyloctanoic, and 4-methylnonanoic acids) that are associated with mutton-like flavor [44]. These vBCFA are harder to detect, especially in meat from lean [24] and young animals (Watkins et al. 2014). In addition, vBCFAs proportions are lower in pasture-fed lambs compared to grain-fed lambs [45]. Therefore, the association between heptadecanoic acid and the vBCFA may explain the negative correlation observed between the proportion of heptadecanoic acid present in total fatty acids and the overall and flavor-liking scores for consumers in cluster 2. Our data show that for consumers in cluster 2, one of the least preferred meat was from PMER-W lambs, which were older and with higher intramuscular fat levels and higher proportions of heptadecanoic acid [13,14]. Following our observation, Prescott, Young and O’Neill [43] observed that Japanese and New Zealand consumers preferred lamb meat with a low rather than high vBCFA content. It should be noted that the overall liking scores for all consumers and for consumers in cluster 2 were positively correlated with the relative abundances of pentane and 6-methyl-5-hepten-2-one. This ketone was previously found to be odor-active in 40 week-old boiled mutton (would be classified as a lamb in New Zealand) [46].

On the other hand, and in contrast to what was observed for consumers in cluster 2, neither overall liking nor flavor-liking scores from consumers in cluster 1 were correlated with PUFA, oleic or heptadecanoic acid. The overall liking scores from these consumers were negatively correlated with octanoic acid, and flavor scores negatively correlated with hexanoic, heptanoic, and octanoic acids. The preference map indicates that the meat products preferred by consumers in cluster 1 were characterized by lower proportions of these acids, which are associated with sour, rancid, fatty, or sweaty notes. Octanoic acid also has been positively associated with goat/mutton-like odor [47,48] or with lamb-flavor [48], indicating that consumers from cluster 1 may prefer products with less intense lamb-flavor. Despite the correlations observed between these short-chain fatty acid compounds and flavor liking and overall liking scores, the lack of differences in their abundance among the different types of lamb meat evaluated suggests that these compounds were not the main drivers of consumer liking in this study.

Hexanal abundance, a major secondary product of linoleic acid oxidation, is frequently used to predict meat lipid oxidation. It has been reported to negatively correlate with meat acceptability in pork [49]. In the current study, hexanal proportions differed among the different types of lamb meat but were not correlated with consumer flavor-liking scores. Consumers from cluster 2 preferred meat with a higher proportion of PUFA. Their overall liking scores were positively correlated with the relative abundances of seven volatile compounds derived from PUFA oxidation, such as hydrocarbons, aldehydes, alcohols, and ketones. The relatively low amount of total fatty acid content in meat from this study may suggest that a desirable meat flavor can result from low abundances of volatiles derived from lipid oxidation, but higher abundances could harm its flavor. Differences in the meat antioxidant content, which is dependent on animal diet, could also have affected producing volatile compounds by modifying the extent of lipid oxidation [50].

Consumers perceived meat flavor variation among different commercial New Zealand lamb types, especially after segmentation according to their overall liking scores. Furthermore, consumers are willing to pay significantly higher prices for better quality, showing a clear differential price opportunity for premium lamb. About half of the consumers preferred products with a specific fatty acid composition. They derived volatile compounds associated with a characteristic flavor of meat from pasture-fed animals. The remaining consumers were less influenced by differences in the fatty acid profile and abundances of derived volatiles except for those associated with sour, rancid, fatty, or sweaty odor descriptors. Consumer willingness to pay more for premium quality lamb and the differences between consumer segments in overall preference for different types of meat support the opportunity to add value to New Zealand lamb through product differentiation and branding.

## Figures and Tables

**Table 1:** Consumer liking scores of grilled lamb loins (m. *Longissimus lumborum*) from 6 typical New Zealand commercial animal groups.

	Commercial Lamb Loin Products ^β^						SEM ^γ^	*p*-Value
	WEAN-W	REDC-W	GRASS-W	CHIC-E	CHIC-W	PMER-W		
**All Consumers ^α^** (*n* = 160)								
Tenderness	70.0 ^ab^	75.6 ^a^	71.4 ^ab^	75.1 ^a^	74.9 ^a^	67.4 ^b^	1.5	<0.001
Juiciness	62.3	64.1	65.8	65.3	64.9	63.1	1.5	0.551
Flavor liking	65.0 ^b^	71.1 ^a^	68.2 ^ab^	68.3 ^ab^	72.2 ^a^	64.3 ^b^	1.4	<0.001
Overall liking	66.9 ^bc^	72.0 ^ab^	69.1 ^abc^	69.3 ^abc^	73.1 ^a^	65.6 ^c^	1.4	0.001
**Cluster 1 ^α^** (*n* = 85)								
Tenderness	70.8	71.3	76.2	69.8	74.2	71.7	1.9	0.141
Juiciness	63.8 ^ab^	61.1 ^b^	70.6 ^a^	61.8 ^b^	65.5 ^ab^	68.6 ^ab^	1.9	0.002
Flavor liking	64.1 ^c^	66.3 ^bc^	75.6 ^a^	62.9 ^c^	71.9 ^ab^	70.2 ^abc^	1.9	<0.001
Overall liking	67.4 ^bc^	66.8 ^bc^	76.6 ^a^	63.2 ^c^	73.2 ^ab^	72.2 ^ab^	1.7	<0.001
**Cluster 2 ^α^** (*n* = 75)								
Tenderness	69.2 ^bc^	80.4 ^a^	65.9 ^c^	81.0 ^a^	75.7 ^ab^	62.6 ^c^	2.2	<0.001
Juiciness	60.5 ^ab^	67.5 ^a^	60.3 ^ab^	69.3 ^a^	64.2 ^ab^	57.0 ^b^	2.2	<0.001
Flavor liking	65.9 ^bc^	76.6 ^a^	59.8 ^cd^	74.2 ^a^	72.6 ^ab^	57.7 ^d^	2.0	<0.001
Overall liking	66.3 ^bc^	77.7 ^a^	60.5 ^c^	76.1 ^a^	72.9 ^ab^	58.3 ^c^	2.0	<0.001

^a,b,c,d^ Different superscript letters denote significant differences between values in the same row according to Tuckey’s test (*p* ≤ 0.05). ^α^ All consumers, cluster 1, and cluster 2: mean scores within each category were estimated using all consumers or consumer groups in cluster 1 and cluster 2 based on their overall liking scores. ^β^ WEAN-W, 4 month-old composite wethers; REDC-W, 6–8 month-old Perendale × LambSupreme wethers finished on red clover; GRASS-W, 6–8 month-old composite wethers finished on grass; CHIC-E, 6–8 month-old composite ewes finished on chicory; CHIC-W, 6–8 month-old composite wethers finished on chicory; PMER-W, 12 month-old Merino wethers finished on pasture. ^γ^ SEM, standard error of LS-means.

**Table 2:** Correlations between overall liking scores and total fatty acid content (*n* = 48) with proportions of selected fatty acids in fresh meat for all consumers and consumers in cluster 1 and cluster 2.

			Pearson’s Correlation Coefficients ^α^ with			
			Overall Liking Scores			Total Fatty Acids
Variable	Mean	SD ^β^	All Consumers (*n*= 160)	Cluster 1 (*n* = 85)	Cluster 2 (*n* = 75)	
**N** (8 per animal group)			48	48	48	
**Mean**			69.2	69.8	68.9	
**SD**			7.0	7.4	10.8	
**Total fatty acids** (FA), mg/100 g fresh tissue	2500	701	−0.21	−0.08	−0.34 *	1.00
**Fatty acids**, % of total FA						
C14:0	2.5	0.9	−0.03	−0.07	0.02	0.16
Iso C15:0	0.1	0.0	−0.10	0.25 ^t^	−0.35 *	0.37 **
Anteiso C15:0	0.1	0.0	−0.05	0.20	−0.11	0.08
Iso C17:0	0.3	0.1	−0.13	0.27 ^t^	−0.39 **	0.21
Anteiso C17:0	0.4	0.1	−0.21	0.20	−0.46 ***	0.57 ***
C17:0	1.0	0.1	−0.15	0.12	−0.41 **	0.46 **
C17:1 *cis*−10	0.2	0.2	0.07	−0.26 ^t^	0.32 *	−0.13
C16:0	21.4	1.5	−0.06	0.00	−0.13	0.59 ***
C18:0	14.9	1.6	0.04	0.20	−0.18	0.12
C18:1 *trans*-11	2.5	0.7	0.16	−0.05	0.39 **	−0.53 ***
C18:1 *cis*-9	32.1	3.7	−0.27 ^t^	0.00	−0.52 ***	0.62 ***
C18:1 *cis*-11	1.0	0.1	0.08	−0.08	0.31 ***	−0.53 ***
C18:2 *n-6*	4.8	1.5	0.23	−0.07	0.51 ***	−0.73 ***
C18:3 *n-3*	2.6	0.7	0.24	−0.16	0.50 ***	−0.60 ***
CLA *cis*-9, *trans*−11	1.0	0.3	0.07	−0.09	0.24	0.12
C20:4 *n-6*	1.5	0.5	0.12	0.08	0.26 ^t^	−0.77 ***
C20:5 *n-3*	1.2	0.4	0.18	−0.13	0.46 **	−0.73 ***
C22:5 *n-3*	1.1	0.2	0.19	−0.07	0.44 **	−0.73 ***
C22:6 *n-3*	0.3	0.1	0.24	0.00	0.39 **	−0.61 ***
BCFA	1.1	0.2	−0.16	0.23	−0.38 **	0.33 *
SFA	41.7	2.0	−0.06	0.14	−0.29 *	0.65 ***
MUFA	37.2	3.4	−0.26 ^t^	−0.03	−0.47 ***	0.66 ***
PUFA	12.6	3.3	0.23	−0.09	0.52 ***	−0.77 ***
PUFA *n-3*	5.2	1.4	0.23	−0.13	0.50 ***	−0.72 ***
PUFA *n-6*	6.4	1.9	0.22	−0.03	0.48 ***	−0.79 ***
*n-6*:*n-3*	1.2	0.2	0.01	0.18	0.04	−0.34 *

* *p* < 0.05; ** *p* < 0.01, *** *p* < 0.001.

**Table 3:** Volatile compounds and their corresponding retention times (Rt), linear retention indices (RI) and their odor descriptor [22,23,24,25,26].

Compound	Rt, min	Calculated RI ^α^	Odor Descriptor
**Alcohol**			
Methanol	5.912	910	Alcoholic
1-Butanol	9.555	1137	Fusel, oily, sweet, balsamic
1-Pente*n-3*-ol	9.807	1153	Green, fruity
1-Pentanol	11.198	1241	Fusel, oily, sweet
Z-2-Penten-1-ol	12.270	1312	Green, fruity
1-Hexanol	12.719	1343	Green, fruity, oily, fusel
1-Octe*n-3*-ol	14.070	1439	Green, mushroom, earthy, oily
1-Heptanol	14.159	1445	Green, woody, fatty, musty, fatty
2-Propyl-1-pentanol	14.631	1480	-
1-Octanol	15.520	1548	Green, waxy, fruity
E-2-Octen-1-ol	16.294	1609	Green, fatty, citrus
2,7-Octadien-1-ol	17.146	1680	-
2-Methyl-1-hexadecanol	18.379	1786	-
1-(2-Butoxyethoxy)-ethanol	18.553	1801	-
**Aldehyde**			-
2-Methyl-butanal	6.249	922	Chocolate, roasted, stink beetle
3-Methyl-butanal	6.299	925	Aldehydic, sweaty, stink beetle
Pentanal	7.174	987	Fermented, fruity, nutty, pungent
Hexanal	8.728	1085	Green, fresh, fatty, aldehydic
Heptanal	10.434	1192	Green, fresh, fatty, aldehydic
Z-4-Heptenal	11.322	1249	Green, oily, fatty, dairy, fishy
Nonanal	13.579	1403	Waxy, aldehydic, green, fresh
Decanal	15.018	1509	Aldehydic, sweet, waxy, green
E,E-2,4-Heptadienal	15.067	1513	Fatty, green, oily, aldehydic
E-2-Nonenal	15.592	1554	Fatty, green, aldehydic
Undecanal	16.376	1616	Waxy, aldehydic, green, fatty
E-2-Decenal	16.946	1663	Waxy, fatty, earthy, green
2-Undecenal	18.229	1772	Fresh, fruity, orange peel
Tridecanal	18.871	1830	Fresh, clean, aldehydic, nutty
Pentadecanal	21.121	2042	Fresh, waxy
**Benzenoid compound**			-
Toluene	8.174	1051	Sweet
Benzaldehyde	15.628	1557	Fruity, strong, sharp
**Furans**			-
2-Ethyl furan	6.777	959	Chemical, sweet, burnt, earthy
2-Pentyl furan	11.096	1234	Fruity, green, earthy, vegetable
**Hydrocarbons**			-
Pentane	3.823		-
E-2-Octene	5.361	844	Sweet, green, floral, burning
Z-2-Octene	5.679	875	Fatty, oil
2,2,6-Trimethyl-octane	6.426	934	-
Decane	7.361	1000	-
**Ketone**			
2-Butanone	6.069	909	Acetone, ethereal, fruity
2-Octanone	11.965	1291	Earthy, herbal, woody, fruity
Acetoin	12.057	1297	Sweet, buttery, fatty, dairy
6-Methyl-5-hepten-2-one	12.742	1345	Citrus, green, musty, cheesy
2-Nonanone	13.490	1396	Fruity, fresh, green, cheesy
2-Decanone	14.923	1302	Orange, floral, fatty, peach
**Organic Acids**			
Acetic acid	14.480	1469	Acidic, sharp, pungent, sour
Butanoic acid	16.700	1643	Acetic, cheesy, buttery, fruity
4-Hydroxy-butanoic acid	17.051	1672	-
Hexanoic acid	19.235	1863	Sour, fatty, sweaty, urine-like
Heptanoic acid	20.390	1971	Rancid, sour, sweaty, cheesy
Octanoic acid	21.498	2079	Fatty, waxy, rancid, oily, cheesy
Nonanoic acid	22.675	2189	Waxy, dirty, cheesy, dairy
**Sulfur compounds**			
Dimethyl sulfide	4.652	760	Sulfurous, onion, cabbage, cauliflower
2-Ethyl-1-hexanethiol	14.631	1480	-
Dimethyl sulfone	20.019	1936	Sulfurous, burnt
**Others**			
N, N-Dibutyl-formamide	18.491	1795	-

^α^ Calculated RI: calculated linear retention index relative to a series of alkanes C7–C30.

**Table 4:** Least square means for the relative abundance of volatile compounds detected in the headspace of grilled lamb loins (*m. Longissimus lumborum*) from six typical New Zealand commercial animal groups and their Pearson’s correlation coefficients with the mean overall liking scores from all consumers (*n* = 160), and from cluster 1 (*n* = 85) and cluster 2 (*n* = 75) consumers.

	Commercial Lamb Loin Products ^α^								Pearson’s Correlation Coef. with Overall, Liking Scores ^β^		
Compound	WEAN-W	REDC-W	GRASS-W	CHIC-E	CHIC-W	PMER-W	SEM ^γ^	*p*-Value ^θ^	All Consumers	Cluster 1	Cluster 2
**Alcohols**											
Methanol ^δ^	0.23 (0.21–0.24)	0.21 (0.19–0.23)	0.25 (0.23–0.27)	0.23 (0.22–0.25)	0.23 (0.21–0.25)	0.26 (0.24–0.28)		0.506	−0.11	0.06	−0.18
1-Butanol ^δ^	0.37 ^a^ (0.34–0.41)	0.22 ^b^ (0.20–0.25)	0.19 ^b^ (0.17–0.21)	0.22 ^b^ (0.20–0.25)	0.23 ^b^ (0.21–0.25)	0.42 ^a^ (0.38–0.47)		<0.001	−0.26 ^t^	−0.07	−0.32 *
1-Penten-3-ol	2.52 ^a^	1.71 ^bc^	1.49 ^c^	1.94 ^b^	1.88 ^bc^	1.49 ^c^	0.16	<0.001	0.04	−0.21	0.14
1-Pentanol	6.38 ^a^	6.90 ^a^	7.08 ^a^	7.37 ^a^	7.32 ^a^	4.84 ^b^	0.47	0.004	0.33 *	0.10	0.39 **
Z-2-Penten-1-ol	0.68 ^a^	0.45 ^bcd^	0.38 ^cd^	0.53 ^b^	0.50 ^bc^	0.36 ^d^	0.05	<0.001	0.06	−0.21	0.22
1-Hexanol	1.53	1.39	1.10	1.31	1.31	1.07	0.14	0.181	0.00	−0.28 *	0.25 ^t^
1-Octen-3-ol	2.44 ^a^	1.64 ^b^	1.70 ^b^	2.08 ^ab^	2.05 ^ab^	1.50 ^b^	0.21	0.034	0.00	−0.12	0.16
1-Heptanol ^δ^	6.31 ^a^ (5.38–6.84)	6.67 ^a^ (6.15–7.22)	6.94 ^a^ (6.40–7.51)	7.13 ^a^ (6.58–7.72)	7.18 ^a^ (6.62–7.77)	4.55 ^b^ (4.20–4.93)		0.002	−0.03	−0.17	0.05
2-Propyl-1-pentanol	0.58 ^a^	0.42 ^b^	0.32 ^b^	0.42 ^b^	0.39 ^b^	0.32 ^b^	0.04	<0.001	0.04	−0.20	0.22
1-Octanol	0.58 (0.52–0.65)	0.48 (0.43–0.53)	0.54 (0.48–0.60)	0.49 (0.44–0.55)	0.51 (0.46–0.57)	0.53 (0.47–0.59)		0.828	−0.06	−0.18	0.03
E-2-Octen-1-ol	0.29 ^a^	0.20 ^b^	0.19 ^b^	0.23 ^ab^	0.23 ^ab^	0.18 ^b^	0.02	0.022	0.01	−0.13	0.19
2,7-Octadien-1-ol	0.15 ^a^	0.10 ^b^	0.08 ^b^	0.11 ^b^	0.10 ^b^	0.07 ^b^	0.01	0.001	0.05	−0.19	0.24
2-methyl-1-Hexadecanol ^δ^	0.08 (0.07–0.10)	0.09 (0.07–0.10)	0.10 (0.09–0.12)	0.09 (0.08–0.11)	0.09 (0.08–0.11)	0.14 (0.12–0.16)		0.193	−0.18	−0.11	−0.14
1-(2-butoxyethoxy)-Ethanol	0.07 ^b^	0.08 ^b^	0.08 ^b^	0.11 ^a^	0.11 ^a^	0.10 ^ab^	0.00	0.02	−0.09	−0.12	0.05
**Aldehydes**											
2-methyl-Butanal ^δ^	0.16 (0.13–0.19)	0.13 (0.11–0.13)	0.13 (0.11–0.13	0.14 (0.11–0.17)	0.11 (0.09–0.14)	0.18 (0.15–0.22)		0.641	−0.03	−0.09	−0.08
3-methyl-Butanal ^δ^	0.30 (0.26–0.34)	0.28 (0.24–0.31)	0.28 (0.25–0.31)	0.29 (0.26–0.33)	0.28 (0.25–0.32)	0.35 (0.31–0.40)		0.731	−0.05	−0.13	−0.07
Pentanal	3.18 ^abc^	2.93 ^bc^	2.90 ^cd^	3.58 ^ab^	3.73 ^a^	2.23 ^d^	0.24	0.001	0.21	−0.09	0.32 *
Hexanal	50.15 ^a^	34.51 ^cd^	38.70 ^bcd^	44.41 ^abc^	45.52 ^ab^	31.24 ^d^		0.006	0.03	−0.10	0.11
Heptanal	8.29	8.34	6.22	8.14	8.11	5.51		0.064	0.08	−0.27 ^t^	0.34 *
Z-4-Heptenal ^δ^	0.80 (0.70–0.81)	0.89 (0.78–1.01)	0.64 (0.56–0.73)	0.80 (0.70–0.91)	0.63 (0.55–0.72)	0.53 (0.47–0.60)		0.082	0.17	−0.16	0.27 ^t^
Nonanal ^δ^	5.79 (5.33–6.28)	5.21 (4.80–5.66)	5.59 (5.16–6.07)	5.94 (5.47–6.44)	5.48 (5.05–5.95)	4.84 (4.46–5.26)		0.676	−0.02	−0.22	0.15
Decanal ^δ^	0.19 (0.16–0.22)	0.17 (0.15–0.20)	0.19 (0.17–0.22)	0.19 (0.17–0.22)	0.24 (0.21–0.28)	0.21 (0.18–0.24)		0.705	0.16	0.07	0.15
E,E-2,4-Heptadienal ^δ^	0.16 ^a^ (0.14–0.18)	0.10 ^c^ (0.09–0.11)	0.09 ^c^ (0.08–0.10)	0.12 ^bc^ (0.10–0.13	0.14 ^ab^ (0.12–0.15)	0.08 ^c^ (0.07–0.09)		0.002	0.12	−0.12	0.18
E-2-Nonenal ^δ^	0.40 ^a^ (0.36–0.45)	0.34 ^abc^ (0.30–0.38)	0.27 ^bc^ (0.24–0.30)	0.32 ^abc^ (0.28–0.35)	0.35 ^ab^ (0.32–0.39)	0.25 ^c^ (0.23–0.28)		0.045	0.05	−0.29 *	0.29 ^t^
Undecanal ^δ^	0.06 (0.05–0.07)	0.05 (0.04–0.06)	0.06 (0.05–0.07)	0.05 (0.04–0.06)	0.07 (0.06–0.09)	0.06 (0.05–0.08)		0.748	0.08	0.02	0.10
E-2-Decenal (E) ^δ^	0.09 (0.07–0.11)	0.07 (0.06–0.09)	0.09 (0.08–0.11)	0.08 (0.07–0.10)	0.10 (0.08–0.12)	0.10 (0.08–0.12)		0.864	0.01	−0.05	0.01
2-Undecenal	0.09 (0.07–0.11)	0.09 (0.08–0.11)	0.12 (0.10–0.14)	0.09 (0.08–0.12)	0.11 (0.09–0.13)	0.10 (0.08–0.12)		0.929	0.02	0.00	0.02
Tridecanal ^δ^	0.07(0.06–0.07)	0.06(0.05–0.06)	0.06 (0.05–0.07)	0.07 (0.06–0.08)	0.07 (0.06–0.07)	0.05 (0.04–0.06)		0.231	−0.02	−0.18	0.21
Pentadecanal ^δ^	0.06 ^ab^ (0.05–0.06)	0.06 ^ab^ (0.05–0.06)	0.05 ^b^ (0.04–0.05)	0.07 ^a^ (0.07–0.08)	0.06 ^ab^ (0.06–0.07)	0.04 ^c^ (0.03–0.04)		<0.001	0.14	−0.13	0.42 **
**Benzenoid compounds**											
Toluene ^δ^	0.25 (0.21–0.31)	0.22 (0.18–0.27)	0.19 (0.16–0.24)	0.16 (0.13–0.19)	0.14 (0.11–0.17)	0.16 (0.13–0.20)		0.321	−0.24	−0.23	−0.13
Benzaldehyde	0.22 ^ab^	0.21 ^ab^	0.19 ^b^	0.27 ^a^	0.26 ^a^	0.17 ^b^	0.02	0.002	0.22	−0.07	0.39 **
**Furans**											
2-ethyl-Furan	0.37 ^a^	0.33 ^a^	0.27 ^ab^	0.37 ^a^	0.33 ^a^	0.16 ^b^	0.04	0.010	0.10	−0.18	0.29 *
2-pentyl-Furan	35.71 ^a^	29.10 ^ab^	29.10 ^ab^	34.65 ^a^	34.40 ^a^	25.17 ^b^	2.38	0.022	0.06	−0.14	0.22
**Hydrocarbons**											
Pentane	0.71 ^bc^	1.12 ^a^	1.02 ^a^	1.01 ^a^	0.92 ^ab^	0.55 ^c^	0.08	<0.001	0.38 **	0.17	0.48 ***
E-2-Octene ^δ^	0.75 ^a^ (0.62–0.90)	0.40 ^bc^ (0.33–0.48)	0.38 ^bc^ (0.31–0.45)	0.22 ^c^ (0.18–0.26)	0.26^c^ (0.21–0.31)	0.44 ^b^ (0.36–0.53)		<0.001	−0.07	−0.13	−0.13
Z-2-Octene ^δ^	0.28 (0.25–0.33)	0.18 (0.15–0.20)	0.26 (0.23–0.30)	0.20 (0.18–0.23)	0.20 (0.17–0.22)	0.21 (0.19–0.25)		0.134	−0.11	−0.01	−0.26 ^t^
2,2,6-trimethyl-Octane ^δ^	0.44 ^a^ (0.33–0.59)	0.01 ^b^ (0.01–0.01)	0.01 ^b^ (0.01–0.01)	0.01 ^b^ (0.01–0.01)	0.01 ^b^ (0.01–0.01)	0.78 ^a^ (0.59–1.04)		<0.001	−0.12	−0.06	−0.14
Decane	0.12 ^ab^	0.05 ^c^	0.07 ^c^	0.07 ^c^	0.08 ^bc^	0.16 ^a^	0.02	0.003	−0.01	0.00	0.02
**Ketones**											
2-Butanone ^δ^	0.36 (0.34–0.39)	0.36 (0.36–0.38)	0.30 (0.28–0.32)	0.32 (0.30–0.34)	0.30 (0.28–0.32)	0.34 (0.32–0.37)		0.201	−0.01	−0.16	0.14
2-Octanone ^χ^	0.05	0.03	0.03	0.03	0.04	0.04	0.27	0.178	0.14	0.00	0.18
Acetoin ^δ^	0.12 (0.07–0.23)	0.43 (0.22–0.85)	0.10 (0.06–0.20)	0.94 (0.50–1.76)	0.47 (0.25–0.89)	0.18 (0.10–0.32)		0.127	0.00	−0.25 ^t^	0.34 *
6-methyl-5-Hepten-2-one	0.41 ^a^	0.36 ^ab^	0.23 ^b^	0.42 ^a^	0.45 ^a^	0.12 ^b^	0.06	0.003	0.35 *	0.03	0.57 ***
2-Nonanone ^δ^	0.04 (0.03–0.05)	0.03 (0.02–0.04)	0.03 (0.02–0.04)	0.03 (0.02–0.04)	0.04 (0.03–0.05)	0.04 (0.03–0.05)		0.705	0.12	0.05	0.15
2-Decanone ^δ^	0.03 (0.03–0.04)	0.02 (0.02–0.03)	0.03 (0.02–0.03)	0.02 (0.02–0.03)	0.03 (0.03–0.04)	0.04 (0.03–0.05)		0.594	0.12	0.07	0.11
**Organic Acids**											
Acetic acid ^δ^	1.23 (1.07–1.43)	1.05 (0.91–1.22)	1.02 (0.88–1.18)	1.28 (1.11–1.48)	1.09 (0.95–1.27)	0.86 (0.74–0.99)		0.442	−0.06	−0.15	0.13
Butanoic acid ^δ^	0.90 ^a^ (0.75–1.08)	1.06 ^a^ (0.88–1.26)	0.69 ^ab^ (0.57–0.82)	0.97 ^a^ (0.81–1.16)	0.88 ^a^ (0.74–1.05)	0.44 ^b^ (0.37–0.53)		0.017	0.06	−0.13	0.29 *
4-hidroxy-Butanoic acid ^δ^^,^^ε^	0.97 ^b^ (0.84–1.11)	1.70 ^a^ (1.48–1.95)	1.08 ^b^ (0.94–1.24)	1.05 ^b^ (0.92–1.21)	1.07 ^b^ (0.94–1.23)	0.54 ^c^ (0.47–0.62)		<0.001	0.23	0.01	0.28 ^t^
Hexanoic acid ^ε^	0.77 ± 0.03	0.66 ± 0.08	0.54 ± 0.06	0.80 ± 0.07	0.82 ± 0.06	0.84 ± 0.07		0.064	−0.06	−0.19	0.13
Heptanoic acid	0.16	0.15	0.14	0.17	0.17	0.16	<0.01	0.161	−0.17	−0.23	0.00
Octanoic acid ^δ^	0.27 (0.25–0.29)	0.24 (0.22–0.26)	0.22 (0.21–0.24)	0.29 (0.26–0.31)	0.26 (0.23–0.28)	0.28 (0.25–0.30)		0.326	−0.29 *	−0.30 *	−0.12
Nonanoic acid	4.35	4.23	4.25	4.48	4.44	4.25	0.10	0.364	0.01	−0.06	0.08
**Sulphur compounds**											
Dimethyl sulphide	0.88 ^a^	0.63 ^bcd^	0.41 ^d^	0.76 ^ab^	0.66 ^abc^	0.45 ^cd^	0.08	0.002	0.17	−0.21	0.35 *
2-ethyl-1-Hexanethiol ^δ^	0.61 ^a^ (0.53–0.70)	0.37 ^bc^ 0.32–0.43)	0.27 ^bc^ (0.24–0.31)	0.40 ^b^ (0.35–0.46)	0.36 ^bc^ (0.31–0.41)	0.25 ^c^ (0.22–0.29)		0.001	0.04	−0.18	0.22
Dimethyl sulfone ^δ^	0.98 ^ab^ (0.84–1.14)	1.35 ^a^(1.16–1.58)	0.68 ^bc^ (0.59–0.80)	0.57 ^c^ (0.49–0.66)	0.60 ^c^ (0.51–0.69)	1.04 ^ab^ (0.90–1.22)		0.001	−0.05	−0.04	−0.03
**Others**											
N, N-dibutyl-Formamide	0.13	0.11	0.10	0.12	0.11	0.09	0.12	0.417	0.22	0.10	0.27

^a,b,c,d^ Different superscript letters denote significant differences between values in the same row according to Tuckey’s test (*p* ≤ 0.05). ^α^ WEAN-W, 4 month-old composite wethers; REDC-W, 6–8 month-old Perendale × LambSupreme wethers finished on red clover; GRASS-W, 6–8 month-old composite wethers finished on grass; CHIC-E, 6–8 month-old composite ewe finished on chicory; CHIC-W, 6–8 month-old composite wethers finished on chicory; PMER-W, 12 month-old composite wethers finished on pasture. ^β^ All consumers, cluster 1, and cluster 2: mean scores within each category were estimated using all consumers or consumers in cluster 1 and in cluster 2 based on their overall liking scores. SEM, LS-means standard error. ^θ^ Symbols for each value denote significant correlations; ^t^ *p* < 0.10; * *p* < 0.05; ** *p* < 0.01, *** *p* < 0.001. 1/x transformation, back-transformed Ls-means are presented. ^δ^ log10 transformation, back-transformed Ls-means with the respective 95% confidence interval within brackets. ^ε^ unequal variance between commercial lamb-meat products, LS-mean ± SEM.

## Data Availability

The data presented in this study are available on request from the corresponding author. Although consumer data have been anonymised, data are not publicly available.
